# Effects of Physical (In)activity on Platelet Function

**DOI:** 10.1155/2015/165078

**Published:** 2015-10-18

**Authors:** Stefan Heber, Ivo Volf

**Affiliations:** Institute of Physiology-Centre for Physiology & Pharmacology, Medical University of Vienna, Schwarzspanierstraße 17, 1090 Vienna, Austria

## Abstract

As platelet activation is closely related to the liberation of growth factors and inflammatory mediators, platelets play a central role in the development of CVD. Virtually all cardiovascular risk factors favor platelet hyperreactivity and, accordingly, also physical (in)activity affects platelet function. Within this paper, we will summarize and discuss the current knowledge on the impact of acute and habitual exercise on platelet function. Although there are apparent discrepancies regarding the reported effects of acute, strenuous exercise on platelet activation, a deeper analysis of the available literature reveals that the applied exercise intensity and the subjects' cardiorespiratory fitness represent critical determinants for the observed effects. Consideration of these factors leads to the summary that (i) acute, strenuous exercise can lead to platelet activation, (ii) regular physical activity and/or physical fitness diminish or prevent platelet activation in response to acute exercise, and (iii) habitual physical activity and/or physical fitness also favorably modulate platelet function at physical rest. Notably, these effects of exercise on platelet function show obvious similarities to the well-recognized relation between exercise and the risk for cardiovascular events where vigorous exercise transiently increases the risk for myocardial infarction and a physically active lifestyle dramatically reduces cardiovascular mortality.

## 1. Introduction

A large body of evidence indicates that both acute exercise and habitual physical activity affect platelet function. This is of special interest as the inflammatory and immunomodulatory consequences of platelet activation are increasingly recognized and platelets therefore seem to be of central importance not only to the final stages of cardiovascular disease (CVD), but also to the development of these diseases. Therefore, a modulation of platelet function by acute exercise and/or habitual physical activity might represent a mechanistic link between physical exertion and its observed effects on CVD. This would be especially interesting as a remarkable strong correlation exists between CVD-related mortality and physical activity, as we will discuss later in this review.

In order to give a structured overview based on currently available literature, the first part of this review will deal with the influence of acute (mostly strenuous) exercise on platelet function. To introduce the reader to different aspects of platelet activation as well as platelet function tests (and also as interpretation of obtained results may critically depend on the applied methodologies), this will be done from a platelet-centered view where different aspects of platelet activation are treated separately. Subsequently, the impact of exercise intensity and the subjects' cardiorespiratory fitness on the effects of exercise on platelet function is summarized and discussed, including the modulating effects of cardiorespiratory fitness/physical activity on platelet function in the resting state.

## 2. Introduction to Platelets and Their Function

Platelets represent the smallest formed elements of blood. They are anucleate cells with a life-span of 7–10 days and contain a variety of intracellular organelles, including different types of secretory granules.

Activation of platelets, finally resulting in platelet degranulation and aggregation, is essential for hemostasis and can be triggered by several specific platelet-stimulating mediators (e.g., thrombin, ADP, and elements of the extracellular matrix) as well as by shear and oxidative stress.

As blood platelets are of central importance to the process of (primary) hemostasis and coagulation, abnormalities in platelet function (resulting in thrombosis or bleeding) result in severe and potentially lethal consequences. In principle, activation of platelets immediately results in platelet aggregation and subsequent thrombus formation—however, these consequences of platelet activation can to some extent be antagonized by functional endothelium, pointing to a crucial role of endothelium-derived mediators that counteract platelet activation (namely, nitric oxide and prostacyclin) in this process. However, as activated platelets compromise endothelial function, the situation is complex.

The importance of platelets to the development of atherosclerotic disease is apparent from the fact that platelet activation is associated with accelerated atherosclerosis and correlates with severity of this disease in humans. An injection of activated platelets exacerbates the formation of native atherosclerotic lesions and an increase in systemic platelet activation has been described for a variety of atherosclerotic diseases including coronary artery disease [1, 2] and cerebrovascular disease [3].

While the in vitro assessment of platelet reactivity in different settings (including acute exercise and training) might include the final consequences of platelet activation (i.e., aggregation), measurement of in vivo platelet activation depends on more subtle parameters.

As different pathways of platelet activation might result in distinct patterns of “platelet activation markers,” such studies require the consideration of several aspects of platelet activation for a final interpretation of obtained results.

## 3. Influence of Acute Exercise on Diverse Aspects of Platelet Activation

### 3.1. Platelet Count and Volume

Several studies show that acute exercise results in a transient increase in platelet count. This increase is caused by hemoconcentration and by platelet release from the liver, lungs, and, importantly, the spleen [4–6]. The latter contracts in response to elevated concentrations of epinephrine, as can be seen with increasing exercise intensity where epinephrine raises from preexercise plasma concentrations of 400–800 pM to >3500 pM with intense exercise [7–9]. Interestingly, platelets stored in the spleen have been reported to be significantly larger than normal circulating platelets [4, 6, 10]. Accordingly, some studies have reported an increase of mean platelet volume in response to exercise [11–13], although this could not be confirmed in all studies [14–16].

### 3.2. Activation of Glycoprotein IIb/IIIa

Glycoprotein IIb/IIIa (GPIIb/IIIa, integrin *α*IIb*β*3) is constitutively expressed on the surface of resting platelets and represents the main fibrin(ogen) receptor on these cells. Upon platelet activation, this receptor undergoes a conformational change resulting in a drastically increased affinity towards fibrin(ogen), enabling the firm binding of platelets to fibrin(ogen) and thus facilitating platelet-platelet interaction, that is, platelet aggregation and the formation of stable thrombi. Only a limited number of studies found an increase in GPIIb/IIIa activation after exercise [11, 17]—most studies reported no such effects [13, 18–22].

### 3.3. Platelet Aggregation

Potential effects of exercise on platelet aggregation have been addressed by a multitude of studies using different techniques. The vast majority of these studies found increased platelet aggregability after exercise in response to various agonists, while a few studies found no such effect [23–26] or even an inhibition of platelet aggregation by exercise [16, 27, 28]. Importantly, platelet aggregation is affected by cell density (see above); therefore, interpretation of experimental results might be difficult, as an increase in agonist-induced platelet aggregation is not necessarily related to platelet hyperreactivity when platelet count is not corrected.

Nevertheless, increased platelet aggregation in response to exercise was observed by light transmission aggregometry without [11, 15, 29–32] and after adjustment of platelet count in platelet-rich plasma (PRP) [21, 33, 34], but also with other techniques including electrical impedance aggregometry [13, 35–41], filtragometry [42–44], calculation of reduction of single (unaggregated) platelets after agonist stimulation [45–50], and platelet aggregation after application of shear stress by a rotational viscometer [20, 51, 52]. Notably, this observed increase of in vitro platelet aggregation after acute exercise is not in contrast to those studies that found no effects of exercise on GPIIb/IIIa activation, as platelet aggregation rather depends on sensitivity of platelets towards agonists than on the basal activation state of glycoprotein IIb/IIIa.

Another test indicative of platelet aggregate formation (following adhesion at high shear) is carried out by the Platelet Function Analyzer (PFA-100), where platelets in whole blood are activated by the combined action of shear stress and agonists, resulting in closure of an aperture. After exercise, shorter closure times have been reported [26, 29, 53]; however, also these results have to be interpreted in light of the fact that this assay can be influenced by platelet count [54], which was increased in two of these three studies after exercise; in the third study [29] no data regarding platelet count have been provided. Decreased closure times in response to exercise were also observed with hemostatometry [55, 56], where defined pressure is applied to drive blood flow through tubing and time until closure due to platelet plug formation is measured after puncture of the tubing.

### 3.4. Platelet Adhesion

Various platelet adhesion assays have been used in the literature to test for an effect of exercise on platelet function. These studies have yielded inconsistent results; whereas one study showed decreased platelet adhesion to fibrinogen (evoked by shear-stress with a rotational viscometer) [20], other groups (using different experimental techniques) did not find any effect [23, 32, 58]; however, results of one study [57] indicate a role of exercise intensity-dependent cortisol levels in this process. Wang et al. used an assay which was designed to test the resistance of adhered platelets against detachment by defined shear stress applied by a flow of buffer. With this method, the authors found more adhering platelets after exhaustive exercise [45, 47–49, 59], indicative of a stronger platelet-surface interaction in response to acute exercise.

### 3.5. Platelet Degranulation

Degranulation represents an integral consequence of platelet activation that results in the concomitant release of a variety of soluble mediators and the expression of distinct proteins on the platelet surface due to membrane fusion of the granules with the platelet membrane. Consequently, de novo protein expression, for example, of P-selectin (CD62P), on the platelet plasma membrane as well as plasma levels of soluble mediators, for example, *β*-thromboglobulin (*β*-TG, CXCL7) and platelet factor 4 (PF4, CXCL4), can be used as a measure of (in vivo) platelet degranulation/activation.

After exercise, several studies found increased plasma levels of *β*-thromboglobulin [30, 31, 37, 45, 60, 61] and platelet factor 4 [27, 45, 60, 62]. As both *β*-thromboglobulin and platelet factor 4 represent very sensitive marker of platelet activation, these findings represent strong evidence that exercise activates platelets, although a small number of studies reported unchanged [26, 63] or decreased [16] levels of *β*-thromboglobulin after exercise.

Although the (activation-dependent) expression of P-selectin is not restricted to platelets, the fact that degranulation initially results in the expression of membrane-bound P-selectin (that is later on cleaved off to form soluble P-selectin) makes this glycoprotein an invaluable marker of platelet activation. P-Selectin represents a pivotal adhesion molecule mediating cell-cell contact of platelets with leukocytes, endothelial cells, and other platelets. Binding of platelet P-selectin to its receptor PSGL-1 on leukocytes results in aggregate formation of platelets with leukocytes (see below), promoting the stimulation of leukocytes—a process involved in many inflammatory conditions.

The influence of acute exercise on the expression of P-selectin on platelets has been addressed by several studies and yielded contradictory results. Basal P-selectin expression (i.e., without intentional platelet stimulation applied ex vivo) has been found to be increased [17, 42, 44, 64–68] after acute exercise, although this was not in all studies statistically significant [13, 18, 21]. Notably, several studies found no influence of exercise on basal P-selectin expression [11, 19, 22, 69–71]. Agonist (including shear stress) induced P-selectin expression after exercise was increased in most studies [17, 42, 44, 51, 66, 69, 70]; but again this effect was not always significant [18], absent [19, 22, 71] or there was even a decrease in P-selectin [20]. Similarly, soluble P-selectin was shown to be increased [21, 49, 72] or unchanged [20, 51, 73] after exercise.

### 3.6. Platelet-Leukocyte Aggregates

Consistent with results of studies showing increased platelet P-selectin expression after exercise, formation of aggregates with (various subsets of) leukocytes (platelet-leukocyte aggregates, PLAs) has been detected in response to acute exercise.

Overall PLAs were found to be increased [42, 69] and studies specifically addressing the involved leukocyte subtypes found increased numbers of platelet-granulocyte aggregates [70, 71], including platelet-neutrophil [16, 22, 44] and -eosinophil aggregates [22] and also platelet-lymphocyte [70] and -monocyte aggregates [44, 67, 70, 71]. Only one single study reported a reduction of platelet-monocyte aggregates after exercise [74].

Within these studies, both basal [16, 42, 44, 67, 69–71, 74] and agonist-induced [22, 42, 44, 69–71] formation of platelet-leukocyte aggregates were determined.

### 3.7. Platelet-Derived Microparticles

A further possible consequence of platelet activation is the release of platelet-derived microparticles (PDMPs) that constitute small (between 0.1 *μ*m and 1.0 *μ*m) membrane vesicles that represent an important link between platelet activation and plasmatic coagulation, as PDMPs provide a large surface of phosphatidylserine necessary for tenase and prothrombinase complex formation. After exercise, the levels of PDMPs in plasma have been reported to be increased [75–77], although some studies could confirm this finding only in response to platelet agonists [70] or to the application of shear stress [78, 79].

### 3.8. Thromboxane A_2_

Platelet activation also involves the release of effectors that are part of positive feedback loops for platelet activation in an auto- and paracrine manner. Besides ADP, a molecule of pivotal importance in this context is thromboxane A_2_, which is synthesized upon platelet activation. Its degradation product thromboxane B_2_ serves as a marker of in vivo platelet activation that can be readily measured. After exercise, elevated plasma [25, 32, 35–37, 42, 66] as well as urinary levels [11, 50] of thromboxane B_2_ have been detected, consistent with a platelet-activating effect of acute exercise.

### 3.9. Platelet Cytosolic Calcium

Modulation of the concentration of cytosolic Ca^2+^ represents a mechanism of central importance to the activation state of platelets. Increased cytosolic free calcium ions constitute a very early event of platelet activation and function as important second messengers in platelets. An increase in cytosolic Ca^2+^ has consistently been reported after exercise [34, 46, 48, 59, 80], although some authors only reported small, nonsignificant effects [38, 50]. Additionally, also agonist-induced Ca^2+^ levels have been found to be increased after exercise [46, 48, 59, 80].

### 3.10. Platelet Inhibiting Pathways

Nitric oxide (NO), prostacyclin (PGI_2_), and CD39/CD73 represent the physiologic effectors of the three major pathways that are relevant to the inhibition of platelet function (for a detailed review see [81]).

(Increased) production and release of both NO and prostacyclin is mediated by an increase in cytosolic calcium concentration, which can be triggered by shear stress (what is obviously relevant to exercise) and a multitude of other mediators. Whereas NO is produced by platelets as well as other cells including endothelial cells and red blood cells, prostacyclin synthesis occurs in endothelial cells and smooth muscle cells but is absent in platelets. Redundancy between these two systems has been reported, where one system takes over when the other one is compromised, for example, increased NO generation when prostacyclin production is impaired [82]. Both NO and PGI_2_ inhibit platelet function via an increase of cyclic nucleotides (NO mainly via cGMP and prostacyclin via cAMP), which subsequently activate their respective protein kinases (cGMP: protein kinase G; cAMP: protein kinase A), which in turn phosphorylate key proteins ultimately leading to platelet inhibition. Notably, since platelet activation is associated with an increase in cytosolic calcium concentration, NO generation within platelets represents also an early consequence of platelet activation, thereby playing an important autoregulatory role that limits excessive aggregation, adhesion, and thrombus growth [83, 84]. Thus, increased intraplatelet amounts of cyclic nucleotides can be the result of (and indicative of) platelet inhibition, for example, by the endothelium (release of NO and PGI_2_), or also a result of platelet activation. Consequently, direct and indirect quantification of NO and PGI_2_ may provide information concerning the production of these compounds but do not necessarily indicate inhibition of platelets as the net effect of activating versus inhibitory stimuli remains obscure without a direct assessment of platelet function.

Due to their short in vivo half-life, both NO and PGI_2_ are commonly quantified by their degradation products nitrite and nitrate (in the case of NO) and 6-keto-PGF_1*α*_ (in the case of prostacyclin).

Available studies indicate increased prostacyclin generation in response to acute exercise [32, 37, 49, 66, 85–88] and increased intraplatelet levels of cAMP [66, 80] indicate that platelets are indeed affected.

Similarly, increased plasma levels of NO degradation products nitrite and nitrate have been observed together with platelet activation [34, 46, 48, 50, 80, 85] after acute exercise. While also red blood cells represent a possible source for NO production in response to exercise [89], the functional relevance of increased levels of NO degradation products critically depends on the bioavailability of nitric oxide. As increased generation of NO might be counterbalanced by its inactivation, for example, by reactive oxygen species, observed levels of NO metabolites do not always indicate biologically active NO. However, increased cGMP levels within platelets [48, 66, 80] (in part accompanied by increased platelet aggregability in response to collagen [38]) have been detected in response to exercise, which strongly argue for an increased bioavailability of NO. Notably, one study also reported unchanged basal but decreased cGMP levels in response to the NO-donor SIN-1 [33] after exercise, which would indicate decreased platelet sensitivity towards NO.

CD39/CD73 constitutes a further pathway that interferes with platelet function. CD39 is expressed on a number of vascular cells, including endothelial cells and platelets [90, 91]. CD39 cleaves ADP and ATP to AMP, consequently limiting platelet activation via P2-receptors. AMP is further broken down by endothelial CD73 to adenosine [92], which inhibits platelets via interaction with their A_2_-receptors and a subsequent increase in cAMP. After exercise, basal as well as ADP-induced CD39 expression on platelets has been reported to be decreased while ADP-stimulation increased CD39-expression before as well as after exercise [21]. This is contrastive to other aspects of platelet function (e.g., expression of P-selectin), where agonists and exercise alter platelet function in a similar manner. Thus, the observed exercise-induced decrease in CD39 expression should be interpreted to represent a mechanism facilitating platelet activation rather than a consequence of platelet activation.

*Short Summary.* Taken together, despite some inconsistency and contradictory results, the majority of available studies indicate that acute exercise exerts a significant influence on virtually all aspects of platelet activation. Although (also) different protocols might partly account for contradictory results found in the literature, results from several studies indicate that the applied exercise intensity might represent a critical determinant for the platelet-activating effects of acute exercise.

## 4. Exercise Intensity and Platelet Function

To test for the hypothesis that exercise intensity affects platelet function, one has to consider that there are several ways to define exercise intensity—each with certain advantages and drawbacks. Although it is beyond the scope of this review to discuss this topic in detail (for a comprehensive overview of different methods to determine and to prescribe exercise intensity the reader is referred to a recent review by Mann et al. [93]), one critical issue has to be mentioned. Most studies dealing with acute exercise and platelet function that are covered within this review defined (or prescribed) exercise intensity as a percentage of maximal oxygen consumption, indeed a very well accepted and widely used strategy. Nevertheless, it is well established that a given percentage of maximal oxygen consumption can result in great interindividual differences in metabolic stress, for example, catecholamine levels and blood pH, which are most likely to influence platelet function during physical exercise. Thus, a comparison of results obtained from different studies is compromised by these limitations.

Nevertheless, a few studies directly compared different exercise intensities with regard to their effects on platelets. These studies provide substantial evidence that exercise intensity indeed represents a critical determinant of platelet activation. Wang et al. [45] compared an incremental exercise test until exhaustion with 30 minutes of continuous exercise at 50–55% of maximal oxygen consumption (VO_2_max). Whereas the 30-minute submaximal trial resulted in decreased platelet adhesion and aggregation (*β*-TG and PF4 remained unchanged), all of these parameters were increased after the incremental test until exhaustion. These results were basically confirmed by a study with similar study design [80], where also cytosolic Ca^2+^ levels were measured. Intraplatelet Ca^2+^—basal as well as after ADP stimulation—was shown to be increased only after the incremental test. Similarly, Chicharro et al. [15] reported that platelet aggregation was unchanged after low intensity running for 30 minutes without an increase in blood lactate levels, whereas running for the same duration leading to a mean lactate concentration of 4.6 mmol/L (at rest: mean 0.9 mmol/L) resulted in significantly increased aggregation in response to ADP. These results are in line with results from another group that found increased aggregability in response to ADP and collagen after an incremental exercise test (bicycle ergometer) until exhaustion, whereas aggregability was largely unchanged after 30 minutes of cycling with an intensity of 60% VO_2_max and even decreased in response to low concentrations of platelet agonist [34]. Cadroy et al. [26] applied 30 minutes of cycling to compare two relatively moderate intensities (50% versus 70% VO_2_max) with respect to their effects on platelet function. These authors did not observe any effects on platelet aggregation but found reduced closure time measured with the PFA-100 in response to epinephrine and ADP. Notably, these effects were more pronounced at the higher intensity. Comparable intensities (corresponding to about 55% and 80% of VO_2_max, resp.) were applied in a study where intensities were defined by means of 90% or 130% of the first ventilatory threshold (an intensity above which lactate accumulation in plasma occurs, but a steady state is still reached during continuous exercise). In this study, hemostatometry readings in terms of increased thrombus formation were only affected by the higher intensity [56].

More recent studies also addressed P-selectin expression and platelet-leukocyte formation. Wang et al. [22] observed more PLAs in response to shear stress, LPS and fMLP after 40 minutes of cycling at 80% of VO_2_max compared to rest, but less PLAs (compared to rest) in response to an exercise intensity of 40% VO_2_max. Notably, there were no stimulatory effects of exercise on PLAs in the absence of agonists. Also Hilberg et al. analyzed P-selectin expression as well as different subsets of PLAs in a study where they compared effects of an incremental step test until exhaustion with those of 45 minutes at 90% of the IAT (individual anaerobic threshold, a threshold concept taking into account the rate of decrease of lactate concentration after the end of an incremental exercise test) [71]. Whereas the amount of PLAs—both basal and after platelet activation by TRAP-6—was increased after both exercise interventions, basal P-selectin expression remained unaffected. However, the incremental exercise test resulted in increased agonist-stimulated P-selectin expression. In a subsequent study, the same authors accounted for the potential confounding effects of different exercise durations between groups (as is the case when comparing, i.e., an incremental test of 10–15 min and a submaximal test of 60 min duration) and applied 45–60 minutes of cycling to compare intensities of 80% and 100% of the IAT with respect to their effects on platelet function. Again, they found increased agonist-stimulated P-selectin expression—albeit no difference between exercise intensities—but also increased formation of platelet leukocyte aggregates, which was significantly more pronounced in response to the higher exercise intensity.

Experiments performed with an ergometer for the upper limbs [35] indicate that a interrelation between exercise intensity and platelet activation is not specific for running or cycling. While TXB_2_ was increased after an incremental exercise test until exhaustion but not after 15 minutes of continuous exercise at 75% of maximal heart rate, platelet aggregation increased in response to both exercise protocols, but this increase was more marked after the incremental test.

## 5. Influence of Cardiorespiratory Fitness on Platelet Function

Besides exercise intensity, also cardiorespiratory fitness, that is, the adaptation to long-term exercise training, might represent a critical determinant for alterations of platelet function in response to acute exercise.

Some studies directly compared the effects of acute, strenuous exercise on platelet function between two groups differing in cardiorespiratory fitness and habitual physical activity, respectively. By and large, results obtained from these studies suggest that low cardiorespiratory fitness results in greater platelet activation after acute exercise. Kestin et al. [18] compared the effects of an incremental exercise test on the expression of several receptors located on the platelet surface between sedentary and physically active healthy volunteers. In sedentary individuals, surface expression of GPIb was downregulated (as is the case after stimulation with thrombin [94, 95]), while CD36 was upregulated (consistent with platelet activation [96]) and the fibrinogen receptor GPIIb/IIIa was significantly more activated (in response to thrombin) after exercise. In contrast, none of these changes could be observed in the fitter volunteers. Consistent with these results, platelets of sedentary volunteers showed higher aggregability and adhesiveness after an incremental exercise test performed on a bicycle ergometer compared with platelets of (significantly, but not much fitter) physically active volunteers [45]. Additionally, the increase of plasma *β*-TG and PF4 tended to be more pronounced in the sedentary group. Coppola et al. [21] also compared sedentary with active subjects and found a significantly elevated number of circulating platelet-platelet aggregates after an incremental exercise test in both groups—however, levels were much higher in sedentary volunteers. Moreover, ADP-stimulated P-selectin expression as well as platelet aggregation was only increased in sedentary volunteers after exercise.

Another recently published study assessed the effect of acute (moderate) exercise on the occurrence and procoagulant activity of platelet-derived microparticles. In untrained as well as highly trained individuals, 90 minutes of cycling at 80% of IAT caused a significant increase in both the number of PDMPs and their coagulant activity. However, after two hours the number of PDMPs remained significantly elevated only in untrained subjects [76]. Such a difference in the amounts of highly coagulant microparticles between sedentary and highly trained subjects in the postexercise phase represents an interesting finding, as there is increased risk for sudden cardiac death within this period and this risk is substantially greater for sedentary individuals [97]. Additionally, it should be mentioned that the chosen exercise intensity of 80% of IAT corresponded to 60% of maximal power output (reached in an incremental test) in trained subjects but only to 45% of maximal power output in sedentary individuals. Consequently, trained subjects performed exercise at a higher percentage of their VO_2_max and their platelets were exposed to a higher shear stress. This is especially relevant in light of the fact that shear stress is able to activate platelets and therefore should be considered when interpreting the results of this study.

In addition, also longitudinal studies have been carried out, where acute effects of exercise on platelet function were assessed before and after a long-term exercise training program. In an early study including only 6 healthy volunteers, 20 minutes of cycling with 70–80% of maximal heart rate was shown to cause a significantly increased slope of ADP-induced platelet aggregation, while 12 weeks of regular exercise training abrogated this response to acute exercise. In line with these findings, Wang et al. [47, 48] observed increased platelet aggregability and adhesiveness in response to an incremental exercise test until exhaustion and this effect was diminished or absent after 8 weeks of regular training. Similarly, the exercise-related increase in intraplatelet calcium was blunted after the training period [48]. Subsequent studies by the same authors confirmed these results also for platelet activation by shear stress [51] and oxidized LDL [46]. Notably, all these effects were reversible after several weeks of detraining.

In a population predestined to be particularly unfit, namely, (untrained) patients with spinal cord injury, El-Sayed et al. [98] observed increased aggregation in response to ADP and collagen after 30 minutes of arm cranking exercise with an intensity as little as 60–65% of peak oxygen consumption. However, after 12 weeks of regular exercise training, acute exercise had the opposite effect, namely, an inhibition of aggregation. Furthermore, Chen et al. [79] recently observed an increase of shear-induced PDMP-formation after an incremental exercise test in sedentary volunteers, an effect that was absent after 4 weeks of regular exercise training.

Taken together, these studies show that regular exercise training mitigates the activating effect of acute, strenuous exercise on platelet function. In addition, a number of studies indicate that long-term exercise training also affects platelet function at rest.

An early study performed in overweight, hypertensive men showed that low intensity training (walking/slow jogging exercise at 45–55% VO_2_max, 5x/week for 45–60 min) for 12 weeks led to a reduction of secondary platelet aggregation in blood that was sampled at physical rest [99]. These findings were confirmed and extended by another study also performed in hypertensive patients by de Meirelles et al. [100], who demonstrated decreased platelet aggregation in response to collagen after a period of regular exercise training (12 weeks, 75–85% of maximal heart rate, 3x/week for 40 min). These effects might be attributed to increased levels of nitric oxide, as L-arginine uptake into platelets, NOS-activity, and cGMP levels were increased after the training period.

Importantly, effects of exercise training on basal platelet function have also been shown in healthy volunteers. Exercise training performed on a bicycle ergometer 5x/week for 30 min over a period of ~8 weeks with healthy, but (initially) sedentary, men or women, respectively (at 60% or 50% VO_2_max), decreased both platelet adhesion and aggregation in response to several stimuli, including oxidized LDL and shear stress [46–48, 51]. Additionally, training decreased intracellular calcium levels and increased cGMP levels within platelets as well as increased NO-metabolites in plasma [48]. Indications that exercise training modulates platelet function via the nitric oxide pathway were also obtained from a study in young, sedentary male volunteers where 20 weeks of ergometer training (60% VO_2_max, 3x/week for 30 min) increased plasma and intraplatelet nitrite/nitrate levels and decreased platelet aggregation in response to collagen and ADP [101]. However, a study performed with 7 spinal cord injury patients was not able to show an effect of arm crank exercise (12 weeks, 60–65% VO_2_max 3x/week for 30 min) on platelet aggregation at rest [98].

Taken together, the majority of available studies conclude that exercise training affects basal platelet function in an antithrombotic manner. Studies performed with patients suffering from peripheral arterial disease indicate that effects on platelet function are apparent after a remarkable short duration of exercise training as 2 training weeks (strength training, 30 min effort-free cycle ergometer, 30 min treadmill walking at 3.2 km/h daily) was sufficient to decrease platelet adhesion to fibrinogen (while showing no effect on platelet aggregation) [102]. In healthy volunteers, no short-term training studies have been performed but it is evident that low intensity training is sufficient to affect platelet function in a favorable way. While only limited data are available on the effects of high volume and/or high intensity training on platelet function, there are indications that basal platelet function might be hyperresponsive in competitive athletes (male cyclists) compared to sedentary controls [103]. Nevertheless, these data have only been obtained with the PFA-100 and therefore might have been affected by the different hematologic profile of professional athletes (i.e., hematocrit), so additional studies are clearly required to address this important point.

In any way, physical activity habits and cardiorespiratory fitness appear to have major impact on platelet reactivity, but it should be kept in mind that platelet function observed in physically active subjects obviously represents the physiologic state; therefore, the conclusion that a sedentary lifestyle and low cardiorespiratory fitness affects platelet function in a prothrombotic, proinflammatory manner would probably describe this issue in an accurate way.

Considering exercise intensity as well as physical activity and fitness, inconsistencies between different studies cited in the first, platelet function centered part of this review can be partly explained. For example, in those studies reporting inhibition of platelet aggregation [16, 27, 28] in response to acute exercise, submaximal, prolonged exercise protocols were applied in trained individuals, whereas the majority of studies reporting increased aggregability prescribed maximal exercise protocols until exhaustion to untrained individuals. Further, a relatively moderate exercise protocol (30 min, 60% VO_2_max) was used in the cited study reporting a decrease in platelet adhesion in response to exercise [20]. Moreover, in contrast to those studies where increased *β*-TG plasma levels were measured after exhaustive exercise, unchanged or decreased levels were only observed after 20–30 min exercise protocols with an intensity not higher than 70% of VO_2_max or maximal heart rate, respectively [16, 26].

## 6. Summary and Conclusion

Available literature that has been summarized in this review indicates that (i) acute, strenuous exercise can lead to platelet activation, (ii) regular physical activity and/or physical fitness prevent platelet activation in response to acute exercise, at least to a certain degree, and (iii) although a remarkable low number of studies have been performed on this topic in healthy populations, habitual physical activity and/or physical fitness also favorably modulate platelet function at physical rest.

Therefore, physical activity habits and cardiorespiratory fitness appear to have major impact on platelet reactivity, but it should be kept in mind that platelet function observed in physically active subjects obviously represents the physiologic state, since endurance exercise—especially running—played a key role in the evolution of the human species [104]. Therefore, the conclusion that a sedentary lifestyle and low cardiorespiratory fitness affects platelet function in a prothrombotic manner would probably describe this issue in an accurate way and this viewpoint is also supported from studies where the training phase was followed by a period of deconditioning.

Obviously, platelet function is directly and indirectly affected by physical (in)activity. In this regard, several mechanisms and cell/tissue types are supposed to contribute to the observed effects. Acute exercise results in increased levels of catecholamines as well as increased shear and oxidative stress, all of which are known to activate platelets. This is especially relevant as artery blood flow and shear rate increase in parallel to exercise intensity [105]. While enhanced shear tends to activate platelets, it also stimulates the endothelial production of nitric oxide. Endothelial nitric oxide affects bypassing platelets (thereby counteracting their activation) as well as vascular smooth muscle cells in the medial layer of the artery. The latter results in vessel dilation and, consequently, increased blood flow at lower shear.

As both NO production and antioxidant defense are increased as a consequence of training, also bioavailability of NO is affected by regular exercise and this would help to explain several effects of physical (in)activity on platelet function even more as low shear rates have been shown to stimulate endothelial ROS generation [106].

Notably, while short-term exercise training enhances production and bioavailability of NO, long-term adaptations taking place in specific vascular regions induce structural changes to the vessel resulting in an increase in lumen diameter, thereby decreasing shear rates while normalizing endothelial NO production [107, 108]. Interestingly, there is evidence that also extreme levels of activity might result in detrimental effects to the cardiovascular system [109], but it is unclear at the moment if this also holds true for platelet function.

While the contribution of platelets to the final stages of CVD is obvious, the central importance of these cells and their activation state to the development of cardiovascular disease is increasingly appreciated as chronic platelet activation and platelet hypersensitivity result in increased liberation of growth factors and proinflammatory mediators. Therefore, it is of special interest that the effects of (both acute and chronic) exercise on platelet function show obvious similarities to outcomes from epidemiologic studies on the impact of exercise on the development of cardiovascular events where it has been shown that vigorous exercise transiently increases the risk for myocardial infarction [110–112]. Approximately 6 to 17 percent of sudden deaths have been shown to occur in association with physical exertion [97] and this risk is considerably lower in populations that perform exercise on a regular base [113]. Further, several studies were able to show a strong, inverse, and independent association between cardiorespiratory fitness and cardiovascular (as well as overall) mortality risk [114–116].

This obvious parallelism poses the intriguing question if the assessment of platelet function in response to acute and habitual exercise might help to predict the beneficial effects of physical activity/fitness on (the development of) CVD. This would offer the possibility of acquiring information from platelet-related studies that is not available from long-term studies with cardiovascular events defined as the primary endpoint. Consequently, this would also allow directly studying and comparing the potential effectivity of different training programs and exercise intensities for primary and secondary prevention of CVD. Although this is speculative at the moment, clearly more research is required on this highly relevant topic as well as on the mechanisms that are involved in the modulation of platelet function by habitual exercise.
